# The Effects of Tamoxifen in Combination with Tranilast on CXCL12-CXCR4 Axis and Invasion in Breast Cancer Cell Lines

**Published:** 2014

**Authors:** Sara Darakhshan, Ali Bidmeshkipour, Kamran Mansouri, Hakhamaneshi Mohammad Saeid, Ali Ghanbari

**Affiliations:** a *Department of Biology, Faculty of Science, Razi University, Kermanshah, Iran.*; b *Medical Biology Research Center, Kermanshah University of Medical Sciences, Kermanshah, Iran.*; c *Cellular and Molecular Research Center, Kurdistan University of Medical Sciences, Sanandaj, Iran.*; d *Fertility and Infertility Research Center, Kermanshah University of Medical Sciences, Kermanshah, Iran.*^*.*^

**Keywords:** Breast cancer, Tamoxifen, Tranilast, Metastasis, Chemokines

## Abstract

It has been reported that CXCL12 binding to CXCR4 induces several intracellular signaling pathways, and enhances survival, proliferation, and migration of malignant cells.

In the present study, we examined the effects of anti-estrogen tamoxifen and anti-allergic tranilast drugs as a single or in combination on invasion by two *in-vitro* invasion assays, wound-healing and matrigel invasion on MCF-7 and MDA-MB-231 human breast cancer cell lines. The mRNA expression levels of CXCR4 and CXCL12 were measured by quantitative real time-RT PCR and CXCL12 protein levels were evaluated by ELISA assay.

The data showed that treatment with tamoxifen and tranilast as a single or in combination resulted in decreased CXCR4 and CXCL12 mRNA and CXCL12 protein expression levels. Both *in-vitro* invasion assays markedly showed synergistic effect of tamoxifen when combined with tranilast drug. Either ER-positive or ER-negative breast cancer cells were sensitive to this combination therapy.

In conclusion, Tranilast increases antimetastatic effect of tamoxifen. The synergistic effect of tranilast is not estrogen dependent; however tamoxifen may sensitize the cells for the action of tranilast. The data also support the importance of the CXCR4/CXCL12 interaction in breast cancer metastasis, and further suggest that CXCR4 and CXCL12 are critical targets for tamoxifen and tranilast in combination or alone.

## Introduction

Metastasis is the main cause of death in women with breast cancer (1, 2). Breast cancer preferentially metastasizes to specific target organs such as liver, lung, bone marrow and lymph nodes. Tumor cell migration and invasion are chemokine-dependent (3). CXCL12 (stromal cell-derived factor-1, SDF-1) is a member of the CXC chemokine family, binds to CXC chemokine receptor 4 (CXCR4)4 that is a transmembrane receptor that belongs to the chemokine receptors family (5).

Furthermore, CXCL12-CXCR4 signaling has been shown to play a role in tumor growth, invasion, angiogenesis and bone marrow cell recruitment (6, 7, 8, 9, 10 and 11). The interaction between CXCR4 and CXCL12 mediates the progression and metastasis of breast and other cancers through a chemotactic effect; in the case of breast cancer, primary tumor cells overexpress CXCR4, whereas preferred organ sites of metastasis (lung, liver and bone) overexpress the CXCL12 ligand (3). Tamoxifen ((Z)-1-{4-[2-(dimethylamino)ethoxy]phenyl}- 1,2-diphenyl-1-butene) is a synthetic non-steroidal anti-estrogenic drug widely used for the treatment of breast cancer (12). Although a majority of patients initially benefit from treatment with tamoxifen, 50% of all metastatic breast cancer patients who receive tamoxifen therapy eventually acquire tamoxifen resistance (13).

Based on the literature, combination therapy with other drugs that helps the activity of tamoxifen, is supposed for conquering this resistance and getting better result from this anticancer drug. Tranilast, N-(3', 4'-dimethoxycinnamoyl) anthranilic acid is an antiallergic drug (14), which has the advantage of having a clinical history in Japan for the treatment of allergy and fibrotic disorders, and has few side effects (15).

Considering the circumstance of CXCR4/CXCL12 axis in breast cancer metastasis, we investigated the effects of tamoxifen and tranilast as a single or in combination on CXCR4 and CXCL12 mRNA and CXCL12 protein expression levels and also on two *in-vitro* invasion models in MCF-7 and MDA-MB-231 breast cancer cell lines.

## Experimental

*Cell culture and treatment*

MCF-7(noninvasive human breast cancer cell line, ER-positive) and MDA-MB-231(invasive and metastatic human breast cancer cell line, ER-negative) were grown in RPMI 1640 media supplemented with 10% (v/v) fetal calf serum(FCS) and penicillin/streptomycin antibiotics. Cultures were maintained at 37 °C in a humidified incubator containing 5% CO_2_.

Tamoxifen (TAM) and tranilast (TRAN) were procured from Enzo Life Sciences and dissolved in dimethyl sulfoxide. Single treatments of TAM were 1, 2, 5, 10, 20 µM and for, TRAN were 10, 20, 50, 100, 200 µM as a single treatment. In the combination treatment, it was used 2 µM of TAM with 10, 20, 50, 100, 200 µM of TRAN.

*Cell viability assay*

MCF-7 or MDA-MB-231 cells were seeded at 10^4^ cells/well in 96-well culture plates. All drug concentrations were tested in triplicate wells and the assays were performed in three separate experiments. After incubation for 48 h at 37 °C and 5% CO_2_, 20 μL of MTT solution (5 mg/mL in PBS) was added to each well and incubated for 4h at 37 °C. The medium with MTT were removed, and 100 μL of dimethyl sulfoxide was added to dissolve formazan crystal at room temperature for 30 min. The optical density (OD) of each well was measured by plate reader at 570 nm.

*In-vitro wound assay*

*In-vitro* wound assay for studying of cell invasion was performed using MCF-7 and MDA-MB-231 cells in cultures that contained TAM and TRAN alone or in combination and vehicle control. Briefly, 2×10^5^ cells/well was seeded on 6-well plates for both cell lines and allowed to achieved confluence. A 1-mm width linear wound was created across the center of each well with a plastic tip. Wounded monolayers were then washed three times with medium to remove cell debris and incubated in the absence or 2 μM TAM, 200 μM TRAN and in combination both for 48 h. Photographs of the wounds were captured on day 0 and again 48 h later under phase contrast microscope. After photography, cells were incubated at 37 °C in a humidified incubator containing 5% CO_2_ in medium containing 2% serum in the absence or doses of drugs for 48 h and allowed to migrate. Experiments were repeated three times, in triplicate for both cell lines.

*Migration assay*

Cell invasion assay were performed by transwell chambers with polycarbonate membrane filters (8 µm pore membrane). 

Transwell filters in 6-well plates were coated with matrigel, hydrated for at least 2 h in the tissue culture incubator with 500 μL serum free RPMI in the bottom and 500 μL in the top of the chamber. 5×10^5 ^MCF-7 or MDA-MBA-231 cells were plated in 500 μL serum-free RPMI on top of chamber, while 2 mL RPMI 10% FBS were placed in the lower chambers. Drugs (2 μM TAM, 200 μM TRAN or combination both) were added to the upper chambers. Cells without any drug were used as control. After Forty eight hours of incubation, the filters were removed, washed twice in PBS and fixed in 10% formalin for 15 min. After fixing at room temperature, the chambers were rinsed in PBS and stained with 0.2% crystal violet for 30 min. After washing the chambers by PBS, the cells at the top of the matrigel membrane were removed by several Q-tips.

Now all cells that remain are the ones that have invaded and made it to the bottom side of the membrane. The number of cells was counted in 10 randomly chosen fields using an inverted microscope and plotted as the percentage of invading cells of the total number of the cells. Three independent experiments each one in triplicate was carried out for both cell lines.

*ELISA assay*

ELISA assay were used to determine the CXCL12 protein levels in MCF-7 and MDA-231 cells. Each of the both cell lines (5×10^4^ cells/well) were plated in 24-well plates in 300 µL of medium. Cell culture supernatants were collected after 48 h of treatment with TAM (2 µM) and TRAN (200 µM) or combination of both drugs. CXCL12 concentration was determined using the Quantikine kit (R and D Systems) according to the manufacturer's instructions.

*Quantitative gene expression analysis of CXCL12 and CXCR4*

For quantitative real-time RT-PCR, total RNA was isolated from each cell line at 48 h after treatment with 2 µM TAM and 200 µM TRAN or combination both by using RNeasy Plus Mini kit (Qiagen). First-strand cDNA synthesis and amplification were done using QuantiTect Reverse Transcription Kit (Qiagen). Real time RT-PCR were performed using human-specific primers to CXCL12 forward: ccatgccgattcttcgaaag; reverse: ttcagccgggctacaatctg (product size: 101 bp), CXCR-4 forward: gccttatcctgcctggtattgtc; reverse: gcgaagaaagccaggatgaggat (product size: 130 bp) and GAPDH forward: actctggtaaagtggatattgttgc; reverse: ggaagatggtgatgggatttc (product size: 162 bp), with QuantiFast® SYBER® Green PCR Master Mix (Qiagen) on an iCycler with a multicolor real-time PCR detection system (Bio-Rad,Hercules, CA). The quantity of CXCR4 and CXCL12 transcripts was standardized by human glyceraldehyde-3-phosphate dehydrogenase (GAPDH). All PCRs were performed in triplicate.

*Statistical analysis*

All assays were performed in triplicate in three independent and separate experiments. For all experiments we used SPSS version17. Statistical analysis was performed using the one-way ANOVA followed by turkey's-test. p-values less than 0.05 were considered statistically significant. 

## Results

*Cytotoxic effect of tamoxifen and TRAN in MCF-7 and MDA-MB 231 breast cancer cells*

**Figure1:**
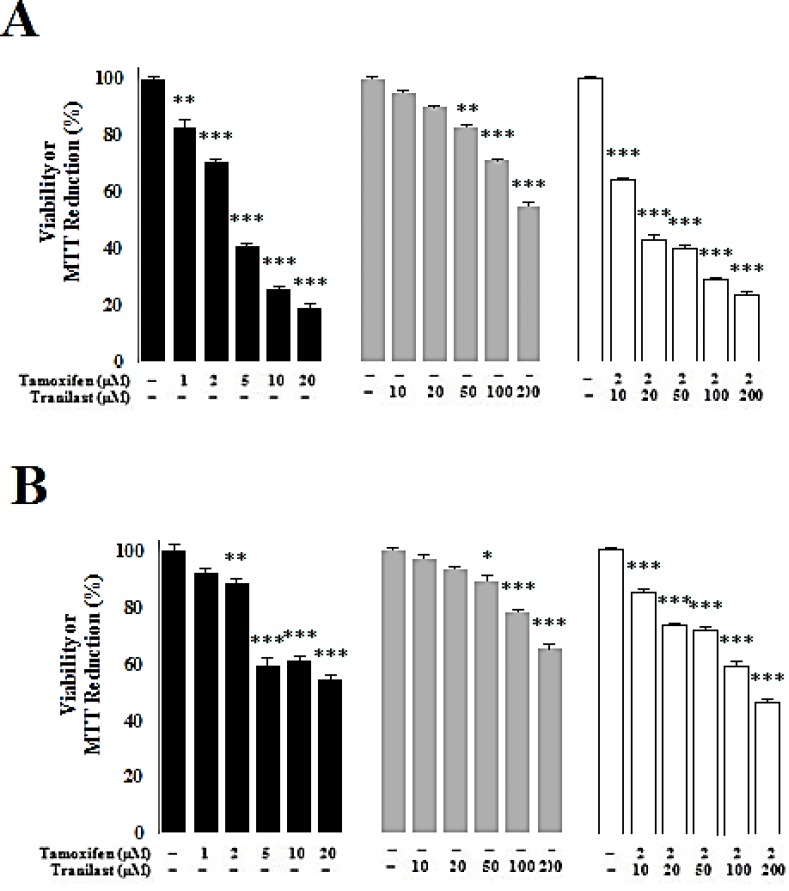
Bar charts represent the effects of different concentrations of TAM and TRAN as a single or TAM in combination with increasing doses of TRAN on cell viability of MCF-7(A) and MDA-MB 231 (B) cell lines.

Cytotoxic effects of TAM and TRAN increased in a dose dependent manner in both MCF-7 (Figure 1A) and MDA-MB 231 cell lines (Figure 1B). In MCF-7 cells, TAM decreased viability by all doses; 1 µM (P=0.03), 2 µM (P=0.02)***, ***5 µM (P=0.00), 10 µM (P=0.00) and 20 µM (P=0.00) while TRAN decreased the viability in the cells only in 50 µM (P=0.04), 100 µM (P=0.03) and 200 µM (P=0.00) (Figure 1A). In MDA-MB 231 cells, TAM decreased viability in 2 µM (P=0.03), 5 µM (P=0.00) 10 µM (P=0.00) and 20 µM (P=0.00) while TRAN decreased the viability in the cells only in 100 µM (P=0.02) and 200 µM (P=0.00) (Figure 1B). TAM (2µM) decreased the viability of MCF-7 cells when combined with 10 µM (P=0.02) and other doses of TRAN (P=0.00) (Figure 1A). In MDA-MB 231 cells, combination of TAM with 10 µM (P=0.04), 20 µM (P=0.03) and other doses of TRAN (P=0.00) decreased the viability (Figure 1B). 

The data of t-test are shown as mean± S.E.M (*p < 0.05; **p <0.01; ***p<0.001).

*Effect of tamoxifen and TRAN on CXCL12 and CXCR4 mRNA levels*

**Figure 2:**
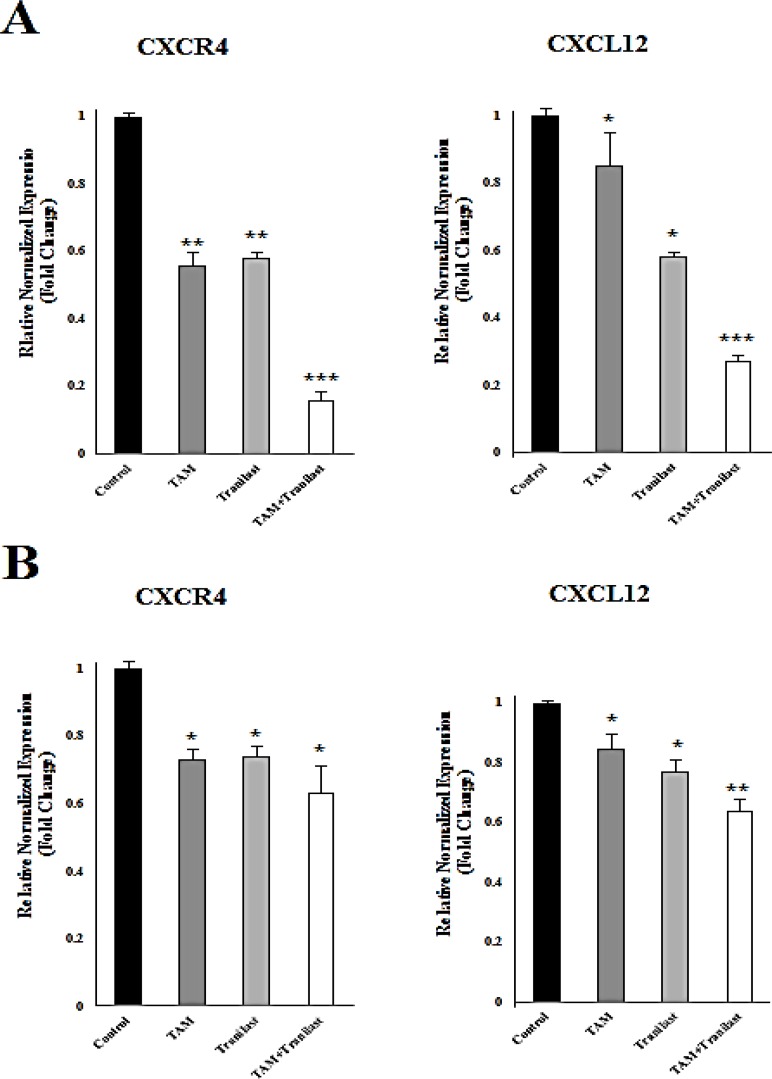
*CXCL12 *and* CXCR4* mRNA were assessed by quantitative real-time RT-PCR after 48h treatment of MCF-7 (A) or MDA-MB-231 (B). Quantitative RT-PCR analysis of CXCR4 and CXCL12 in MCF-7 and MDA-MB-231 cells showed that TAM and TRAN as a single or in combination effectively decreased the expression of CXCR4 and CXCL12 mRNA. The real-time PCR results were normalized against the internal control *GAPDH* and are expressed as a percentage of control cells. The data of t-test are shown as mean± S.E.M (*p < 0.05; **p <0.01; ***p<0.001).

To determine the effects of TAM (2µM) and TRAN (200 µM) on diminution in mRNA levels of CXCR4 and CXCL12 in MCF-7 and MDA-MB-231 cells, mRNA levels in both the cells lines were examined after treatment using quantitative real-time RT-PCR (Figure 2A, B).

In MCF-7 cells, the expression of CXCR4 and CXCL12 was reduced in TAM, TRAN and TAM+TRAN (P=0.00) treated groups (Figure 2A). Also, in MDA-MB-231 cells the expression of CXCR4 and CXCL12 was reduced in TAM (P=0.04) and TRAN(P=0.3) and TAM+TRAN (P=0.00) treated groups (Figure 2B).

These results clearly indicate that, treatment with tamoxifen and TRAN as a single or combination resulted in a decrease in CXCL12 and CXCR4 mRNA level, however this reduction was somehow higher in MCF-7 breast cancer cells. These data indicate that tamoxifen may induce primary effect on expression of these two chemokines and then TRAN induces its synergistic effect.

*Effects of tamoxifen and TRAN on secreted CXCL12*

**Figure 3:**
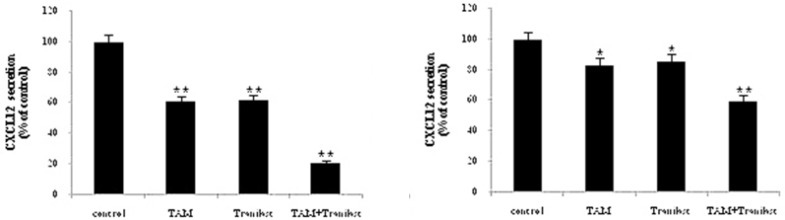
Secreted CXCL12 protein levels after treatment with TAM and/or TRAN for 48 h were determined by ELISA and the values were normalized relative to the total protein concentration. MCF-7 cells (A) MDA-MB-231 cells (B). Each bar is the mean of the three separate experiments.

This effect was also confirmed at the protein level using ELISA assay. We analyzed secreted CXCL12 protein in the culture medium on MCF-7 or MDA-MB-231 cells treated with 2 µM TAM and 200 µM TRAN (48 h) alone or combination by ELISA and the values were normalized relative to the total protein concentration (Figure 3).

The data of t-test are shown as mean± S.E.M (*p < 0.05; **p <0.01; ***p<0.001).

We found that treating MCF-7 cells with TAM (P=0.00) and TRAN (P=0.00) as a single or combined (P=0.00) treatment decreased secreted levels of the CXCL12 protein (Figure 3A). In MDA-MB-231 cells, the protein level of CXCL12 also reduced in TAM (P=0.00) and TRAN (P=0.00) and combined (P=0.00) treated groups (Figure 3B). As shown in Figure 3, combination of TAM and TRAN induces more reduction in the secretion of CXCL12 by the breast cancer cells. The minimum protein levels were observed as an effect of combination treatment. Also, results revealed that inhibition effects of TAM and TRAN as a single or in combination was significantly higher in MCF-7 cells (Figure 3A) than in MDA-MB-231 cells (Figure 3B). The later data support the data of real time- RT PCR and show that TAM may have primary negative effect on migration of breast cancer cells. 

*Effects of tamoxifen and TRAN on migration activity of MCF-7 and MDA-MB 231 cells in-vitro*

**Figure 4:**
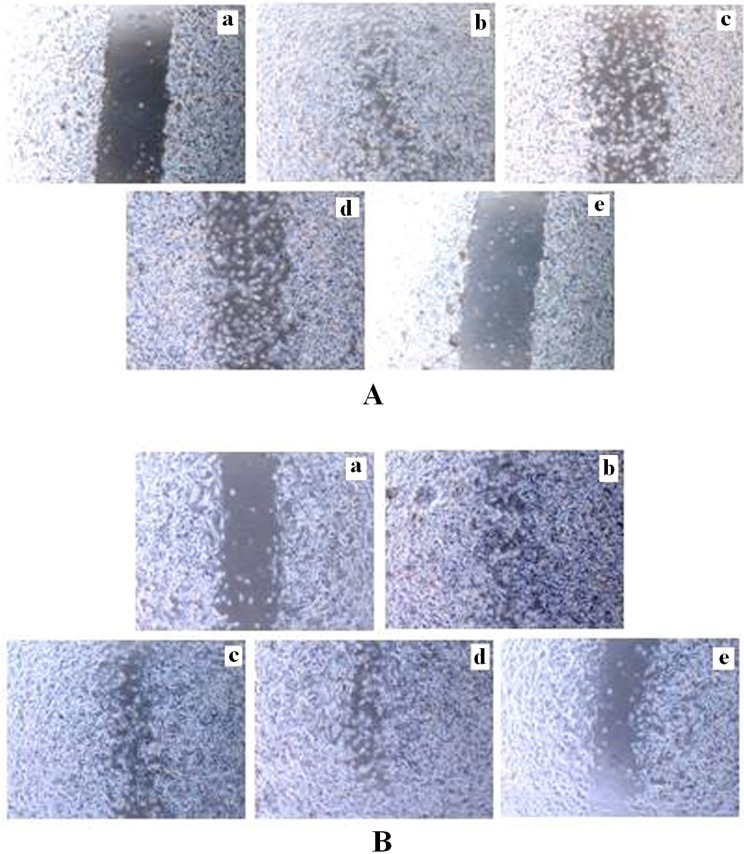
Inhibition of invasion in monolayer wound healing model induced by TAM and TRAN or in combination alone on MCF-7(A) and MDA-MB-231(B) cells. Phase micrographs of cells were taken at various times after monolayer wounding. a The control group in 0 day; b The control group after 48 h; c cells treated with 2 µM TAM ; d cells treated with 200 µM TRAN ; e cells treated with combination

Cell motility was a measurement of metastatic potential of cancer cells. After 48 h treatment, cells in the control group efficiently spread into the wound area to such an extent that the wound boundary was almost indistinguishable, whereas very a few cells in TAM or TRAN treated group spread forward in MCF-7 (Figure 4A; a, b,c,d)and MDA-MB 231 cells (Figure 4B; a, b,c,d). 

The spread of both MCF-7(Figure 4A; e) and MDA-MB 231(Figure 4B; e) cells were more reduced in combination treatment.

*Effects of tamoxifen and TRAN on invasive potential of MCF-7 and MDA-MB 231 cells in-vitro*

To explore whether the invasion activity was inhibited in MCF-7 and MDA-MB 231 cells in response to TAM and TRAN alone or in combination, we examined the invasion activity using insert chamber.

**Figure 5:**
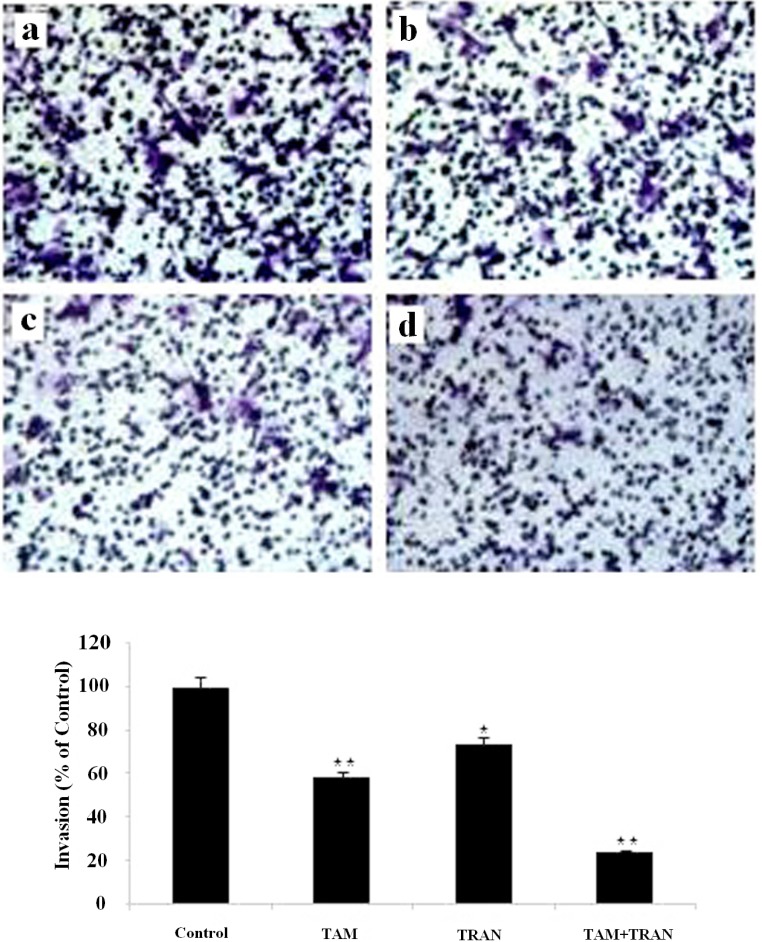
Inhibition of invasion in Matrigel invasion assay induced by TAM and TRAN or in combination alone on MCF-7 cells. (A) Phase micrograph of invading MCF-7 cells. a The control group; b MCF-7 cells treated with 2 µM TAM; c MCF-7 cells treated with 200 µM TRAN; d MCF-7 cells treated with combination (B)Quantification of cell invasion shown in (A). The data of t-test are shown as mean± S.E.M (*p < 0.05; **p <0.01; ***p<0.001).

**Figure 6:**
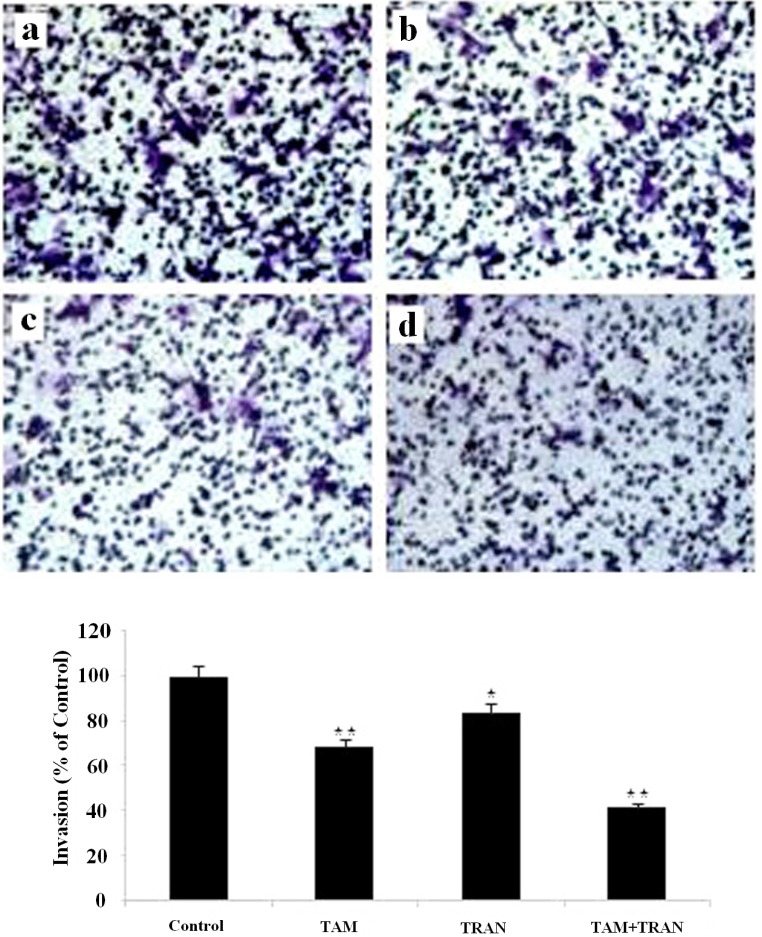
Inhibition of invasion through Matrigel invasion assay induced by TAM and TRAN or in combination alone on on MDA-MB-231 cells by TAM and TRAN alone or in combination. TAM, TRAN and combination inhibits MDA-MB-231 cell invasion through Matrigel. (A) Phase micrograph of invading MDA-MB-231 cells. (a) The control group; (b) MDA-MB-231 cells treated with 2 µM tamoxifen; (c) MDA-MB-231 cells treated with200 µM TRAN; (d) MDA-MB-231 cells treated with combination. (B) Quantification of cell invasion.

In cell migration assay, our data showed that MCF-7 cells and MDA-MB 231 cells in combined treatment decreased the ability to migrate through Boyden chamber by 75 (P= 0.003) and 60% (P= 0.007) (Figure 5, 6).

The data of t-test are shown as mean± S.E.M (*p < 0.05; **p <0.01; ***p<0.001).

## Discussion

Tumor cell migration and invasion are chemokine-dependent. Muller and colleagues^3 ^have provided new insights into potential mechanisms related to organ-specific metastases of breast cancer cells directly related to a CXC chemokine. They found that CXCR4 was the most highly expressed chemokine receptor in human breast cancer. The ligand for CXCR4, CXCL12 mRNA, exhibited peak levels of expression in organs that are preferential destinations of breast cancer metastasis.

The significance of the CXCL12/CXCR4 signaling axis in breast and other tumor progression and invasiveness has been reported by many investigations (6, 16, 17 and 18). CXCL12 regulates the homeostasis, angiogenesis, proliferation, survival and migration of cancer cells (16, 19 and 20). The high expression of CXCR4 has been often associated with an invasive and migratory phenotype of cancer cells (21, 22). CXCL12 interacts with the CXCR4 receptor to initiate several downstream effectors and intracellular signalling pathways that promote migration, invasion, adhesion and proliferation of various cancer cells (3, 16, 23 and 24). 

Tamoxifen (TAM) is a nonsteroidal selective estrogen receptor modulator widely used in the chemotherapy of breast cancer (25). Nonetheless, innate or acquired resistance to tamoxifen is a critical problem for anti-estrogen therapy (26, 27). As a result, there is a need to develop new drugs for combination therapy for the management and treatment of this disease.

In addition to its anti-allergic effects (14), studies have shown that TRAN is effective in reducing the growth of the primary tumour, as well as significantly reducing metastases to the lungs and liver in a mouse model (28, 29).

In human models, studies have suggested that CXCR4 expression could correlate with human epidermal growth factor receptor (HER)-2 expression in breast cancer patients (30), and could be associated with the development of lung metastases (30), liver metastases (31), or bone marrow micrometastases (32, 33) in breast cancer. Kang *et al*. (34) have shown that CXCR4 is overexpressed in MDA231 subclones that present a higher ability to metastasize to bone. Another study suggests that CXCR4 expression in the primary breast tumor is associated with a higher risk for bone metastasis (35). Previous studies have shown that blocking the CXCR4 receptor decreased breast cancer cell invasiveness *in-vitro* and also the reduced of metastases in animal models (3, 17, 36, 37, 38, 39, 40). Boimel *et al*. (41) reported that increased expression of CXCL12 by breast cancer cells can lead to enhanced *in-vivo* invasion. Also, Overexpression of CXCL12 increased *in-vitro* invasion and migration of human breast cancer MDA-MB-231 cells (17). Recently, it has been reported that CXCL12 concentration in blood plasma exhibits a strong association with the risk of distant metastasis of breast cancer cells and patient survival (42).

We used combined treatment of TAM and TRAN to inhibit CXCR4 and CXCL12 protein and mRNA expression levels and determined the effects of these drugs on breast cancer metastasis. We found that the TAM and TRAN as a single or combination of TAM and TRAN significantly inhibited the invasion of the breast cancer cell line MDA-MCF-7 and MDA-MB-231 in wound-healing assay and in Matrigel invasion assay *in-vitro* and that this correlates with decreased invasion in the tumor cells. Further, the present study shows that treatment with TAM and TRAN as a single or in combination inhibited CXCR4 mRNA expression levels and CXCL12 expression at protein and mRNA expression levels in MCF-7 and MDA-MB-231cells, suggesting that TAM and TRAN as a single or in combination may suppress metastases by down-regulating the expression of CXCR4 mRNA expression levels and CXCL12 at protein and mRNA levels breast cancer cells. 

In conclusion, tranilast affects CXCL12/ CXCR4 axis that lead to suppress invasion of breast cancer cells. Metastasis inhibition effect of TRAN is not depending on estrogen receptor; however, TAM may sensitize ER-positive breast cancer cells for the action of tranilast. 

Because of the effectiveness of TRAN on metastasis inhibition of both ER-positive and ER-negative cells, we believe that the information provided in this article suggest suitable combination therapy for future clinical trials for breast cancer.
